# The Altruism Prioritization Engine: How Empathic Concern Shapes Children’s Inequity Aversion in the Ultimatum Game

**DOI:** 10.3390/bs15081034

**Published:** 2025-07-30

**Authors:** Weiwei Wang

**Affiliations:** School of Education and Psychology, Southwest Minzu University, Chengdu 610225, China; wei_psy281@swun.edu.cn

**Keywords:** empathic concern, fairness, inequity aversion, children

## Abstract

Children are not only concerned about fairness but also care for others. This study examined how experimentally induced empathic concern influences children’s responses to inequity, particularly when fairness considerations may conflict with empathy-driven motivations. A sample of 10- to 12-year-old children (*N* = 111, 62 boys, 49 girls) from China were randomly assigned to an empathic or non-empathic condition and completed multiple rounds of ultimatum and dictator games, acting as recipients and proposers. The results showed that children in the empathic concern condition were more likely to accept disadvantageous offers (*F* (1, 109) = 10.723, *p* = 0.001) and reject advantageous offers (*F* (1, 109) = 11.200, *p* = 0.001) than those in the non-empathic condition. Furthermore, in the dictator game, children in the empathic condition shared significantly more resources with the same protagonist (*F* (1, 109) = 110.740, *p* < 0.001). These findings suggest that empathic concern affects children’s inequity aversion and that empathic concern takes priority in guiding children’s inequity aversion when it conflicts with the fairness criterion. Moreover, our findings suggest that altruistic motivations potentially play a role in children’s responses to their inequity aversion.

## 1. Introduction

Inequality and disparities in access to resources are pervasive societal issues. Children also encounter inequalities, and the development of their understanding of fairness is critical for their moral and social growth (Gevaux et al., 2020). While much research has demonstrated that individuals’ responses to unfairness vary depending on social and contextual cues (van Prooijen, 2013), less is known about how such responses are shaped by variations in empathy—particularly in children. The present study addresses this gap by examining the effects of experimentally induced empathic and non-empathic concern, as contextual factors, on children’s responses to both disadvantageous and advantageous inequality.

### 1.1. Empathy and Empathic Concern

Empathy, broadly defined as the ability to understand and share the feelings of others (Hoffman, 2000), encompasses the capacity to affectively share, recognize, understand, and reflect upon the emotions and mental states of others (Bernhardt & Singer, 2012). This involves the ability to take someone else’s perspective and see the world from their point of view. As a core component of social cognition and emotional intelligence, empathy facilitates deeper connections between individuals and is associated with positive social and emotional outcomes (Weisz et al., 2021). Empathy comprises both cognitive and affective dimensions (Davis, 1983; Hall & Schwartz, 2018), and among these, empathic concern—also referred to as compassion—is an emotional and motivational state characterized by a desire to help others and promote their well-being (Bernhardt & Singer, 2012; Light et al., 2015). It involves being emotionally moved by another’s suffering and feeling motivated to act in response (Haidt, 2003). This other-oriented emotional response, which we adopt as our working definition of empathic concern in the present study, typically arises when perceiving another’s need or distress. Consequently, empathic concern plays a vital role in fostering social understanding, improving communication, and enhancing supportive relationships (Gilbert, 2021; Petrocchi et al., 2023). In the present study, we adopt the term empathic concern to refer to the other-oriented emotional response elicited by perceiving another’s need or distress. While closely related to compassion, which encompasses a broader motivational and attitudinal stance (Gilbert, 2014), empathic concern offers a more precise and measurable construct for examining situational emotional responses in individuals.

Because “Emotions prepare for action” (Scherer, 2005), empathic concern is a powerful motivator for actions intended to benefit others, such as offering comfort, sharing resources, or volunteering (Decety & Cowell, 2015; Eisenberg et al., 1994, 2006; Green et al., 2018). This emotional response is a fundamental moral sentiment that encourages ethical conduct and strengthens community bonds (Goetz et al., 2010; Zaki, 2018). Notably, research indicates that individuals, including children, often help others even when it involves some sacrifice on their part. For example, preschoolers will spontaneously help strangers without expecting any reward (Warneken & Tomasello, 2006). Furthermore, children aged three to eight have been observed sharing their resources with others (Benenson et al., 2007) and even choosing to give up some of their own possessions to penalize unfair behavior from others (Jordan et al., 2016; House et al., 2013). These patterns of costly behavior appear across different cultures (Blake et al., 2015), suggesting that the tendency to act for the benefit of others, sometimes at personal expense, emerges early in development and is fostered by social experiences.

Why do people—and children in particular—help at a cost to themselves? One perspective suggests that individuals sometimes help others primarily due to a prosocial orientation. This orientation involves helping to lessen their own negative feelings when witnessing someone else’s distress, with helping serving as a way to alleviate personal discomfort (Hoffman, 1982; Stevens & Taber, 2021). Alternatively, an altruistic orientation offers a different explanation. It posits that empathy directly increases the likelihood of helping behavior motivated by a genuine concern for the other person (FeldmanHall et al., 2015). Observing someone else in distress evokes empathic concern, which then motivates an individual to help alleviate that distress, with the primary goal of benefiting the other person (Batson et al., 1989). In this context, empathic concern can drive helping actions with the ultimate goal of alleviating another’s suffering (Gilbert, 2014; Goetz et al., 2010). The question of whether children’s costly acts of assistance stem from a prosocial or an altruistic orientation remains an area of discussion. For example, students who choose not to apply for a scholarship to allow a classmate in greater need to receive it demonstrate a sacrifice of personal interest for the benefit of another. Such actions might be driven by a genuine desire to alleviate the classmate’s hardship (altruistic orientation) or by a wish to reduce the student’s own feelings of discomfort upon learning of the classmate’s need (prosocial orientation). Regardless of the precise underlying motivation, empathic concern clearly plays a significant role in prompting actions that benefit others, even when those actions involve a personal cost.

### 1.2. The Development of Fairness and Inequity Aversion

Fairness—the expectation that resources and opportunities should be allocated without bias—is a foundational principle guiding social interaction (Brosnan & de Waal, 2014; Tyler, 2000). Far from being a late-developing social norm, recent work shows that the seeds of fairness emerge remarkably early in life. For example, infants as young as four months demonstrate expectations of equal resource distribution (Buyukozer Dawkins et al., 2019) and show intention-based evaluations of distributive actions (Geraci & Surian, 2023). By seven months, infants integrate emotional cues and social roles when judging aggressive versus prosocial acts, evidencing an early-emerging empathic sensitivity (Geraci et al., 2024). Collectively, these findings indicate that fairness evaluations are rooted within the first year of life. The contemporary perspective on early moral development suggests that even infants possess foundational aspects of a sense of justice, including expectations of impartiality and agency in resource distribution (Surian et al., 2025).

As children progress through middle childhood, their understanding of fairness becomes increasingly sophisticated, incorporating considerations of intentions, needs, and social contexts (Elenbaas, 2019). Toddler studies further reveal an incipient intergroup lens: 20- to 30-month-olds preferentially attribute fair behavior to out-group distributors, suggesting early coalition-building motives (Geraci et al., 2024). At four years of age, children demonstrated a notable ability to make prospective judgments and were more likely to apply immanent justice reasoning to predict positive outcomes following good actions (Geraci & Cancellieri, 2024). When observing an allocation between others, six-year-old children may forgo some self-interest to punish an unequal protagonist (McAuliffe et al., 2015). This behavior is referred to as inequity aversion, defined as individuals’ negative response to unequal compensation for equal work (Fehr & Schmidt, 1999). Specifically, people tend to respond negatively both to receiving less than others (disadvantageous inequity aversion, DI) and to receiving more than others (advantageous inequity aversion, AI) (Fehr & Schmidt, 1999). Developmental studies show that DI typically emerges around age four, whereas AI appears later, around age eight (Blake & McAuliffe, 2011). These reactions—both DI and AI—are considered indicative of a concern for fairness (Brosnan & de Waal, 2014). Shaw et al. (2016) suggest that children’s responses to inequity follow different developmental trajectories depending on both the type of inequity and the surrounding context. It is widely argued that advantageous inequity aversion provides stronger evidence of genuine fairness concerns, as it requires individuals to reject outcomes that benefit themselves in order to uphold equity principles (Blake et al., 2015; McAuliffe et al., 2013; Shaw & Olson, 2013).

Two broad classes of evidence have been used to demonstrate that people are averse to inequity: their negative reactions to inequity and their costly rejections of it (Shaw et al., 2016). According to this, one research paradigm that has been extensively used to investigate inequity aversion is the ultimatum game (UG), which has proven to be a robust tool for studying fairness-related decisions in both adults and children (Güth et al., 1982; Falk et al., 2003; McAuliffe et al., 2013). In this game, one participant (the proposer) is given a resource (e.g., tokens or money) and decides how to divide it between themselves and the second player (the recipient). The recipient then chooses to either accept or reject the offer. If the offer is accepted, the proposed allocation is implemented. If rejected, both players receive nothing (Güth et al., 1982). The UG simulates real-life interpersonal interactions by presenting participants with a social exchange involving asymmetrical power and fairness-related decision-making. The proposer controls the division of resources, while the recipient must evaluate the fairness of the offer and choose to accept or reject it. This mirrors real-world scenarios such as negotiating salaries, resolving group resource allocations, or responding to perceived unfairness in peer interactions. The UG has been widely used in developmental research to assess fairness sensitivity and strategic behavior in children. Research using the UG has consistently revealed the widespread phenomenon of inequity aversion in decision-making, where individuals are willing to forgo personal gains to reject unfair outcomes (Fehr & Schmidt, 1999). Thus, recipients’ rejection can be interpreted as an aversion to inequity. Studies have extended these findings to children, demonstrating that even school-aged participants exhibit rejection patterns sensitive to fairness norms, proposer intent, and social context (e.g., Castelli et al., 2014; Jin et al., 2020; McAuliffe et al., 2013). These findings confirm the continued utility of the UG as a tool to study the development of fairness-related decisions in childhood. For example, McAuliffe et al. (2013) investigates 4- to 9-year-olds’ responses to unfair allocations in the UG. By age eight, children begin to reject not only disadvantageous but also advantageous offers (McAuliffe et al., 2013). These findings offer important insights into children’s moral development, suggesting that fairness-based reasoning and social concern are present from an early age and continue to evolve throughout middle childhood.

In addition to the UG, the dictator game (DG) is another widely used paradigm for studying fairness behaviors. Unlike the UG, where recipients can reject unfair offers, the DG allows the proposer (dictator) to allocate resources unilaterally, with no consequences for recipients—they simply receive the amount allocated by the proposer. Thus, the DG is considered a more direct measure of altruistic motivation (Benenson et al., 2007; Fehr et al., 2008). Sharing behavior in the DG reflects a willingness to forgo personal gain to benefit others without external pressure. Combining both the UG and the DG allows researchers to distinguish strategic rejection from genuine concern for others, an approach particularly useful in understanding children’s fairness-related decision-making and motivations (Schug et al., 2016).

### 1.3. The Role of Empathic Concern in Inequity Aversion

Theories suggest that aversion to inequity arises from spontaneous emotional reactions to the distress of the disadvantaged recipient (Hoffman, 1990). Importantly, empathic concern has been proposed as one mechanism that sensitizes individuals to others’ needs (Batson et al., 1991). In the UG, individuals are influenced not only by material self-interest but also by concern for the well-being and intentions of others involved in the interaction (Liu et al., 2018, 2020). This emotional response, driven by empathic concern, often leads to actions aimed at restoring fairness or alleviating suffering (Goetz et al., 2010).

While much of the existing literature on inequity aversion and empathic concern focuses on adults, it is reasonable to expect that similar mechanisms are at play in children. Research reveals that children’s behavior often involves not only equality (equal shares) but also equity considerations, such as merit- or need-based distributions (Paulus, 2014). Given this, understanding how situationally induced empathic concern specifically affects children’s responses to inequity becomes crucial for deepening our understanding of the social–emotional underpinnings of fairness reasoning in development. Specifically, empathic concern may influence children’s aversion to inequity in the UG through two potential psychological mechanisms. Firstly, a prosocial orientation might drive children to reject unfair offers to alleviate their own distress upon witnessing the proposer’s (potential) disappointment or need. Secondly, an altruistic orientation, stemming from empathic concern for the proposer’s well-being, might motivate them to reject offers they perceive as unfairly low for the proposer, even if the offer benefits themselves. Given these possibilities, understanding how situationally induced empathic concern specifically affects children’s responses to both advantageous and disadvantageous inequity in the UG becomes crucial for deepening our understanding of the social–emotional underpinnings of fairness reasoning in development. In the present study, we aim to investigate this by using contextual cues related to the proposer’s need to induce state-based empathic concern in children and examine its effect on their responses to unequal outcomes in the UG.

### 1.4. The Present Study: Examining the Impact of Empathic Concern on Children’s Inequity Aversion Using the Ultimatum Game

While previous studies have examined children’s responses to inequality (e.g., Blake & McAuliffe, 2011; McAuliffe et al., 2013), and more recently, He et al. (2022) demonstrated that empathy modulates people’s fairness-related decisions, important questions remain. Specifically, few studies have explored how experimentally induced empathic concern alters children’s inequity aversion within the UG, nor have they clearly distinguished whether rejections of advantageous inequality stem from genuine altruistic concern or self-focused distress reduction. To address these gaps, the current study examined whether situationally induced empathic concern alters children’s reactions to both disadvantageous and advantageous inequity and whether these UG decisions predict sharing in a subsequent DG. This method allowed us to examine whether empathic concern not only increases costly rejection of unfair offers but also promotes altruistic giving in a no-consequence setting, thereby revealing underlying motivational mechanisms. Children aged 10 to 12 years were selected for this study because this developmental period is critical for the emergence and consolidation of fairness understanding (e.g., Blake & McAuliffe, 2011). At this stage, children begin to show more sophisticated moral reasoning and are able to engage in complex social decision-making tasks such as the UG. Furthermore, this age range is appropriate for understanding how contextual empathic concern influences fairness-related decisions, as younger children may not yet have developed the cognitive and emotional capacities necessary for such tasks. To ensure the feasibility and appropriateness of the experimental procedures and tasks, a pilot study was conducted with a smaller sample of children in the same age range. The pilot confirmed that children could understand the game rules and the empathy manipulation effectively, and that the tasks were engaging and age-appropriate.

Children were randomly assigned to one of two conditions: an empathic concern condition and a non-empathic control condition. In both conditions, children acted as recipients, deciding whether to accept or reject proposals made by a proposer (represented by an animated avatar). In the empathic concern condition, the proposer was portrayed as a disadvantaged character in need of help; in the non-empathic concern condition, the proposer was described without any such contextual cues. Based on existing theories and empirical findings, we proposed the following hypotheses. H1: Children in the empathic concern condition will be more likely to accept disadvantageous offers, reflecting increased tolerance toward inequity when empathic concern is activated. H2: Children in the empathic concern condition will be more likely to reject advantageous offers, indicating heightened concern for fairness and altruistic motivation.

Additionally, we sought to explore the underlying motivation for rejecting advantageous inequity. In the present study, after the UG, participants assumed the role of the dictator in the DG, allowing us to examine their behavior in relation to their decisions in the previous game. By comparing children’s sharing behavior in the DG to their decisions in the UG, we aimed to infer whether their responses in the UG were motivated by altruism or by self-oriented distress regulation. If the motivation for rejection in the UG was to reduce personal distress, differences in behavior would only be observed in the UG rejection rates. This led to the third hypothesis (H3): Children in the empathic condition not only reject advantageous allocations but also share more resources with their partner in the DG. Their behavior is driven by altruism toward the person they empathize with.

Exploring these questions enhanced our understanding of how momentary empathic concern shapes children’s fairness decisions and uncovered the motivational pathways—strategic versus altruistic—underlying early inequity aversion. These insights can inform educational interventions aimed at fostering empathy-driven fairness in childhood social contexts.

## 2. Materials and Methods

### 2.1. Participants

A total of 120 children aged 10 to 12 years were recruited from two public primary schools located in the south of China (Sichuan province), characterized by a diverse socioeconomic population. The schools were selected through collaboration with the local education bureau, aiming to recruit a representative sample of children from the city’s middle-income families. Nine children (six boys and three girls) were excluded due to insufficient valid trials, resulting in a final sample of 111 participants (62 boys and 49 girls, 10- to 12-year-old (*M* age = 11.27, *SD* = 0.31)). Participants were randomly assigned to either the empathic concern condition (*n* = 61) or the non-empathic concern condition (*n* = 50), with a roughly equal gender distribution across groups. G*Power v.3.1 was used for sample size estimation (Faul et al., 2009). According to the analysis (*f* = 0.25, *α* = 0.05, *β* = 0.80, ANOVA: repeated measures ANOVA, between factors, number of groups: 2, number of measurements: 2), a total sample size of 98 participants was required to detect a reliable effect size, which was met by the present study. Gifts were given to each participant as a reward in the study (e.g., erasers, pencils and notebooks). Written informed consent was obtained from parents or guardians prior to the study. The Institutional Review Board of SWUN University approved all the procedures.

### 2.2. Design

We used a 2 × 2 design with the condition (empathic concern or non-empathic concern) as a between-subject design and the inequity type (disadvantageous inequity or advantageous inequity) as a within-subject design. Each child participated in three blocks (totaling 27 trials). There was a five-minute rest between two blocks. The order of disadvantageous unequal, advantageous unequal and equal trials within a block was randomized beforehand and marked on a live-coding sheet.

### 2.3. Procedure

Participants were seated comfortably in front of a computer screen in an electrically isolated room. The experiment began with instructions and a set of UGs for practice trials.

The instruction: “Hi! Welcome to the game, you will play this game with several unknown children who are your age and come from another school. Each unknown game partner will play with you just once. When you make a decision (Yes or No), the game is over, and you will meet another new game partner.”

Children were asked if they had any questions about the words. The instructions continued if no questions were asked.

The instruction: “When a game begins, your game partner will receive 10 coins and he/she will decide how to allocate these coins with you. You can make a decision ‘accept’ or ‘reject’. If you accept, you can say ‘Yes, I’ll take it!’ Both of you will be given the coins according to the allocation. But if you say ‘NO!’, neither of you gets any coins. All the coins in this round will be withdrawn by the computer system.”

Next, the participants received three practice trials. The experimenter provided feedback during each practice trial (e.g., “You got 5 coins in this game”). After the practice trials, the participants were informed that they could not receive their coins during the online game but would receive a present based on the number of coins collected, which they could take with them at the end of all the games.

After all the instructions and practice trials, participants were randomly assigned to either the empathic concern condition or the non-empathic concern condition. In the empathic concern condition, the participants were informed that their game partner was an underprivileged student living in extreme poverty and participants were shown descriptive text (e.g., “Children who grow up impoverished often lack the food, sanitation, shelter, health care, and education they need to survive and thrive”) and images illustrating a typical day in the life of impoverished students. These materials aimed to evoke empathic concern among the participants. In the non-empathic concern condition, the proposer was characterized as a student attending an urban school. Participants were instructed to read the text about their one-day school life. Subsequently, participants were evaluated for empathic concern and took part in the UG and DG.

Empathic concern. To assess empathic concern, an Empathic Response Questionnaire (including three items) was formulated based on the empathic concern scale by Davis (1983). The empathic concern items in the context of the poor student read, “I feel empathic concern for the students suffering from the poverty”; “I am quite touched by the students suffering from the poverty”; and “I have concerned feelings for the students suffering from the poverty.” Responses were given on a 7-point Likert scale ranging from strongly disagree to strongly agree. It was used to measure the extent to which participants felt sympathetic (Pfattheicher et al., 2019).

The result showed high internal consistency reliability among the three items of the Empathic Response Questionnaire (Cronbach’s α = 0.89), and the average of the three scores was calculated as the empathic concern score. It was revealed that children’s scores in the empathic concern condition were significantly higher than in the non-empathic concern condition. The underprivileged protagonist context really induced the empathic concern of participants.

Inequity aversion. Each participant was instructed to play the UG nine times in one block (three blocks in total). In each game, children’s unknown partner acted as the proposer. They were given 10 coins and offered the chance to makes an allocation proposal for how to divide this amount between themselves and the participants. The participants acted as recipients and then were given the choice of accepting or rejecting the proposal. Each block contained nine trials, with three disadvantageous allocations (8–2, 2 coins for the participant), three advantageous allocations (2–8, 8 coins for the participant) and three equal allocations (5–5, 5 coins each). If the participant accepted the proposal, both of them received the amounts allocated by the proposer. However, both received nothing if participants rejected the proposal. Immediately after each UG, participants played a DG with the identical proposer from that UG interaction. Participants were given another 10 coins and had the opportunity to share coins between themselves and their game partners in any manner they wanted. Children were instructed: “Now you will decide how to share 10 coins between yourself and the same boy/girl you just played with. Whatever you give will be taken from your coins.” Participants allocated resources by moving a slider on screen, confirming their choices with a “Submit” button.

The participants’ earnings were represented by coins and calculated in the following parts. For both the empathic and non-empathic conditions, 10 rounds of the UG were randomly selected from the accepted allocations, and the corresponding amounts were accumulated and added to the participants’ base compensation of 30 coins for participating in the experiment. All the earned coins could be exchanged for gifts after the experiment (e.g., 80 coins = an eraser, 90 coins = a notebook, 100 coins = a cup). This incentive structure implied that participants aiming to maximize their compensation would need to reject disadvantageous allocations and accept only advantageous ones. This setup enabled us to distinguish whether participants accepted disadvantageous allocations due to altruistic motives or self-interest. Additionally, it allowed us to examine whether participants would forgo their own interest maximization to pursue fairness by observing their decisions to accept or reject equal allocations. Overall, this manipulation provided a comprehensive framework for analyzing participants’ behavioral motivations. The experimental design was also intended to ensure that participants believed their choices directly influenced their actual compensation, thereby encouraging realistic decision-making.

### 2.4. Data Coding and Analysis

The participant’s actions in each trial (accept or reject) in the UG and the number of coins they shared with their game partners in the DG were coded live by the experimenter for all the sessions. Data analysis was conducted using R software (version R 4.4.1; R Core Team, 2024). To find the differences between the empathic concern and non-empathic concern conditions, the decision data of the participants were analyzed using analysis of variance (ANOVA).

## 3. Results

A repeated measures ANOVA on the empathic concern scores revealed that participants’ scores in the empathic concern condition were significantly higher than in the non-empathic concern condition, *F* (1, 109) = 63.773, *p* < 0.001, *η_p_*^2^ = 0.532.

**Figure 1:**
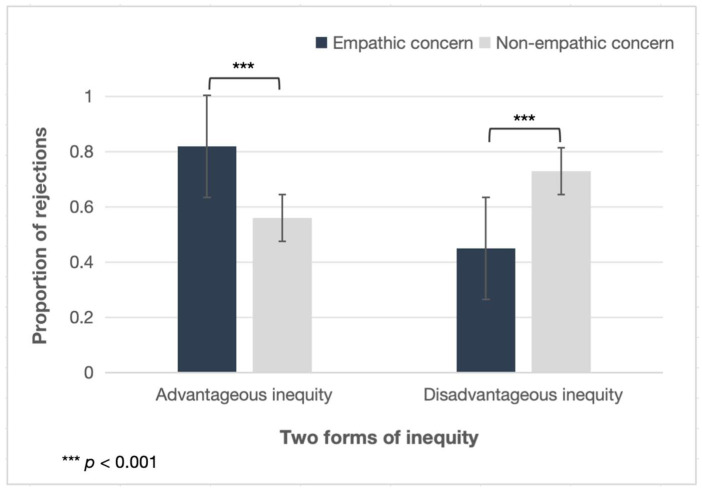
Mean percentages of offers rejected by children aged 10–12 years in the empathic concern and non-empathic concern conditions across the two forms of inequity (advantageous and disadvantageous).

In the UG, the number of rejections in each block of trials was summed as the dependent variable for each participant. The results showed that the rejection rate of children in the empathic concern condition was higher than that in the non-empathic concern condition for the advantageous allocation, *F* (1, 109) = 11.200, *p* = 0.001, *η_p_*^2^ = 0.093. Children in the empathic concern condition rejected more advantageous allocations (*M* = 0.82, *SD* = 0.36) compared with that in the non-empathic concern condition (*M* = 0.56, *SD* = 0.44), while the opposite result occurred in the inequity form of the disadvantageous allocation. The rejection rate of children in the empathic concern condition was lower than that in the non-empathic concern condition, *F* (1, 109) = 10.723, *p* = 0.001, *η_p_*^2^ = 0.090. Children in the non-empathic concern condition rejected more disadvantageous allocations (*M* = 0.73, *SD* = 0.38) compared with that in the empathic concern condition (*M* = 0.45, *SD* = 0.46) (see Figure 1).

**Figure 2:**
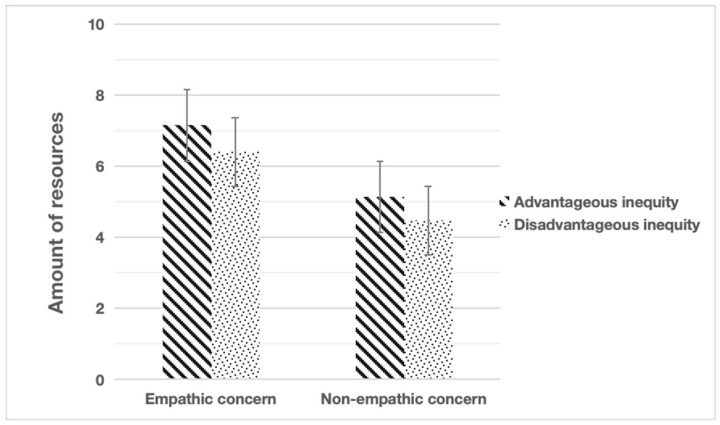
Mean amount of resources shared by children in the dictator games.

In the DG, the number of resources that children shared was summed as the dependent variable for each participant. A one-way analysis of variance was conducted to compare the amount of resources allocated to their game partners by children, *F* (1, 109) = 110.740, *p* < 0.001, *η_p_*^2^ = 0.304. Children shared more resources with the protagonist in the advantageous allocations (empathic concern condition, *M* = 7.15, *SD* = 1.78; non-empathic concern condition, *M* = 5.14, *SD* = 1.14) and in the disadvantageous allocations (empathic concern condition, *M* = 6.41, *SD* = 2.33; non-empathic concern condition, *M* = 4.48, *SD* = 1.15) (see Figure 2).

## 4. Discussion

The present study investigated the influence of experimentally induced empathic concern on children’s responses to inequity in the UG and further explored the underlying motivations through a subsequent DG. Our findings provide valuable insights into the interplay between empathy, altruism, and the development of fairness preferences in children aged 10 to 12 years.

### 4.1. Empathic Concern Shapes Children’s Inequity Aversion

The results demonstrate that inducing empathic concern significantly impacted children’s inequity aversion in the UG. Specifically, children in the empathic concern condition exhibited a greater tendency to accept disadvantageous offers and reject advantageous offers compared to those in the non-empathic control condition. The increased acceptance of disadvantageous allocations suggests that when empathic concern is activated, children may be more willing to tolerate receiving less than a fair share, potentially prioritizing the needs or situation of the proposer.

This is further supported by the heightened rejection rates of advantageous offers in the empathy condition, indicating a concern for the proposer receiving what might be perceived as an unfairly small portion, even if the participant themselves would benefit from accepting. These findings align with the broader literature suggesting that contextual factors can significantly modulate individuals’ responses to unfairness (Pfattheicher et al., 2019) and extend this understanding to the role of empathy in children’s decision-making within the UG. The experimental manipulation, where the proposer was described as a student living in extreme poverty in the empathic concern condition, likely played a crucial role in eliciting this other-oriented response, thereby influencing their fairness preferences in both receiving and rejecting offers.

### 4.2. Altruistic Based Empathic Concern in Children’s Inequity Aversion

An important question that warrants exploration is whether children’s rejection behaviors in the empathic concern condition are motivated (a) by prosociality or (b) by altruism. The empirical findings of the present study support the latter, as empathic-concern-based inequity aversion aligns with altruism, a primary motivator for children’s fairness behaviors. This finding suggests that empathic concern prioritizes altruistic motivations over fairness when confronted with disadvantageous inequity.

A key finding of this study is the crucial role of altruistic motivation underlying the observed influence of empathic concern on children’s inequity aversion. The pattern of the results—increased acceptance of disadvantageous offers and increased rejection of advantageous offers, coupled with the subsequent behavior in the DG—strongly suggests that altruism is a significant driver of these responses. The greater willingness to accept disadvantageous offers in the empathic concern condition implies that children may have been motivated by a desire to help the proposer, even at a cost to themselves.

More compellingly, the increased rejection of advantageous offers suggests an altruistic concern for the proposer receiving a fair share, indicating that children were not solely motivated by self-interest or a simple aversion to any form of inequality. Furthermore, the observation that children in the empathic concern condition shared significantly more resources with the same protagonist in the DG provides direct evidence of an underlying altruistic motivation. In the DG, where there are no strategic incentives, the act of sharing reflects a genuine concern for the well-being of the other individual.

This link between empathic concern, UG responses, and DG sharing behavior supports the notion that children’s decisions in the empathy condition were driven by an altruistic orientation toward the person with whom they empathized, rather than merely a prosocial desire to alleviate their own potential distress at witnessing perceived unfairness. This underscores the idea that empathic concern, as a potent motivator for other-regarding behavior (Decety & Cowell, 2015), guides children’s inequity aversion by prioritizing altruistic considerations, even when they conflict with principles of strict fairness.

### 4.3. Limitations and Future Research

While our study provides valuable insights into the role of situationally induced empathic concern in children’s inequity aversion in the UG, it is important to acknowledge certain limitations.

Firstly, an important consideration is the role of the interaction partner’s identity. In our study, children interacted with an anonymous player represented by an avatar. Children’s behavior, including their experience and expression of empathic concern, as well as their fairness judgments, might differ depending on whether they are interacting with an adult or a peer. Secondly, individual differences among the participants, such as their values and personality traits, could have influenced the relationships observed in this study. For instance, children with a stronger baseline tendency toward empathy or those who hold particular beliefs about fairness might respond differently to the experimental manipulation. Future research could incorporate measures of these individual characteristics to examine their potential moderating effects on the relationship between induced empathic concern and inequity aversion. Finally, our study provides a snapshot of the responses at a specific point in time. Longitudinal research could investigate how the relationship between empathic concern and inequity aversion develops over time.

Several avenues for future research could build upon the findings of this study. One promising direction would be to explore these potential differences by manipulating the perceived identity of the interaction partner and to investigate the role of individual characteristics in the relationship between empathic concern and children’s inequity aversion. Furthermore, to enhance the generalizability of our findings, future research should also investigate this phenomenon in other populations, including children from different cultural backgrounds and geographical locations. This would help to determine the extent to which our results, obtained from a specific sample in China, can be applied more broadly.

## Data Availability

The datasets generated and/or analyzed during the current study are available in the Zenodo, accessed on 14 March 2025. Weiwei, W. (2025). Influence of empathic concern on children’s inequity aversion [Data set]. Zenodo. https://doi.org/10.5281/zenodo.15025353.
